# The study of surface states in a semi-infinite crystal

**DOI:** 10.1038/srep08679

**Published:** 2015-03-02

**Authors:** Huiping Wang, Tingting Gao, Ruibao Tao

**Affiliations:** 1 State Key Laboratory of Surface Physics and Department of Physics, Fudan University, Shanghai 200433, China; 2 Collaborative Innovation Center of Advanced Microstructures, Nanjing 210093, China

## Abstract

An infinite three dimensional (3D) crystal can be constructed by an infinite number of parallel 2D (hkl) crystal planes (CPs) coupled to each other. Based on lattice model Hamiltonian with the hopping between the nearest neighbor (1NN) CPs and all possible neighbor hoppings within each CP, we analytically prove that a (hkl) cut crystal will not accommodate any surface states if the original infinite crystal has the reflection symmetry which results in the forward transfer matrix *F* to be equal to the backward one *B*, named as *F-B* dynamical symmetry. We also study the effect of the longer range couplings among the *n*NN (*n* > 1) CPs and surface relaxation on our conclusion and find that the small perturbation from both factors has no effect on our conclusion based on the perturbation theory. Thus our model may have the potential for studying surface states in some cut crystals with low-index surfaces. Our result may be helpful to visually predict which cutting direction in some non-topological crystals is unfavorable to generate surface states.

Edge/surface and interface states for 3D and 2D crystals, possessing some novel physical properties, have been attracting considerable attention in recent decades. Many interesting and prominent physical phenomena are tightly related to the existence of edge or surface states, such as quantum Hall effect^1^,^2^, quantum spin Hall effect^3^,^4^,^5^,^6^, topological insulator (TI)^7^,^8^,^9^,^10^,^11^,^12^, topological superconductor (TSC)^13^,^14^,^15^,^16^,^17^,^18^ and topological Anderson insulator (TAI)^19^,^20^. Gapless edge or surface states that exist in TIs result from the spin-orbit coupling (SOC), which is highly attractive in recent studies. Besides the interests in the topological states, the study of surface states emerging in some crystals, such as ferroelectric ABO_3_ crystals^21^ and some semiconductors (i.e., Si, Ge), has also been focused on for a long time due to their potential applications. The presence of surface states provides a low dimensional (LD) surface band to be filled with some mobile electrons. If the surface band is partially filled, the mobile electrons in the surface band can strongly couple with the surface softened phonon modes, which may result in the surface reconstruction such as Si and Ge (111) surfaces^22^,^23^. Therefore, if one could have a near-perfect surface with the small relaxation, it would be better to find an appropriate direction to cut the crystal and expect that no surface states emerge. Some ab initio calculations for ABO_3_ Perovskite (001) surfaces have shown that there are no surface states and the surface relaxations are small^24^,^25^. Existence/absence of surface states may greatly change surface physics properties. Thus the study of surface states in such non-topological insulators and semiconductors as well as semi-metals (i.e., graphene) is quite important due to their significant applications. Surface states created in such semiconductors and insulators can also induce some new physical phenomena. As is well known, the electric conduction along domain surfaces and domain walls in ferroelectric materials has recently attracted intense studies^21^,^26^ due to the possibility of creating and controlling some nano-scale 1D/2D conductive paths in wide band gap insulators. For many of them, the SOC does not play a key role and can be negligible. It is also shown that the existence/absence of surface states is sensitive to the cutting surfaces. Theoretical studies have reported the existence/absence of surface states based on some simple models with a few electron modes (orbits) per unit cell and the 1NN and/or 2NN hopping^27^,^28^,^29^. We would extend the model to be more general and closer to some real materials that have no strong SOC and/or strong correlation effect. For experimentalists, it would be useful if there is an intuition tool that can qualitatively tell which cutting direction can be unfavorable or favorable for generating surface states. This work may give insight into the underlying relationship between the existence/absence of surface states and the crystal symmetry. We will start from a general model Hamiltonian at the level of single-particle approximation. In the following, we will prove that there should not be any surface states in (hkl) cut crystals with the hopping between the 1NN CPs when the original infinite crystal has the reflection symmetry for every (hkl) CP which means the forward hopping matrix *F* to be equal to the backward one *B* (*F = B*). For the conventional materials without SOC, if the structure arrangement of all parallel crystal planes has the reflection symmetry, it does satisfy the dynamical symmetry *F = B*.

As is well known, the strong 2NN coupling is favorable to the existence of surface states. However, for some crystal families such as some conventional insulators or semiconductors with negligible SOC as well as semi-metal (i.e., graphene), the 1NN coupling can be dominant if the Miller index (hkl) of the cutting surface is low. But the 2NN and even much longer range couplings can be compatible with (even larger than) the 1NN one for some materials with high-index (hkl) surfaces. In this paper, we will prove a conclusion (“theorem”) for the model with the hopping between the 1NN CPs. However, the *n*NN (*n* > 1) hopping among CPs is able to result in the presence of surface states, which we will discuss in the later section, and the surface decoration may be a good way to manipulate surface states. Based on the perturbation theory, we further demonstrate that the weak longer range hopping and small surface relaxation have no effect on our conclusion.

## Results

### Model Hamiltonian

In general, an infinite 3D crystal can be described by an infinite number of parallel 2D CPs which are periodically arranged one by one with coupling. The direction of CPs can be denoted by Miller index (hkl) where h, k and l can be arbitrary integers. A semi-infinite crystal with the (hkl) cutting surface is called as the (hkl) cut crystal. Firstly we only take into account the model with the hopping between the 1NN CPs, where all possible neighbor hoppings within each CP are included. It would be a reasonable model to some crystals with low-index surfaces where the coupling between the 1NN CPs can be dominant and the couplings among the *n*NN (*n* > 1) CPs are much weaker than that between the 1NN CPs. For a semi-infinite crystal, its each CP is still a crystal with the lower dimensionality. Thus, the Fourier transformation can be applied to each CP since the wave vector 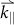 within each CP is a good quantum number. Taking the diagonal representation of the Hamiltonian for each CP, the effective Hamiltonian can be described as follows:
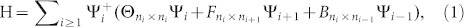
where 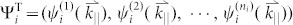 and 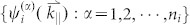 is the second quantized Fermionic wave function of the *α^th^* electron mode in the *i^th^* crystal plane. 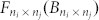 represents the *n_i_* × *n_j_* forward (backward) hopping matrix from the plane *P_i_* to its 1NN CP *P_i+_*_1_(*P_i−_*_1_) and (*n_i_,n_j_*) can be any finite positive integers. 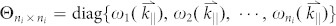 and the boundary condition is {Ψ*_i_* = 0 : *i* < 1} From now on, we omit the symbol 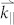 for simplicity. The above Hamiltonian is general and each unit cell of every CP contains many atoms (and maybe different) and each atom can also contribute many different atomic orbits. Thus the model Hamiltonian is reliable for some crystals with low-index surfaces. The conclusion is phrased as follows:

### Conclusion (Theorem)

Based on the above model Hamiltonian with the hopping between the 1NN CPs and all possible neighbor hoppings within each CP, any low index (hkl) cut crystal with negligible SOC will not allow any surface states if the original infinite crystal has the reflection symmetry for every (hkl) CP.

**Figure 1:**
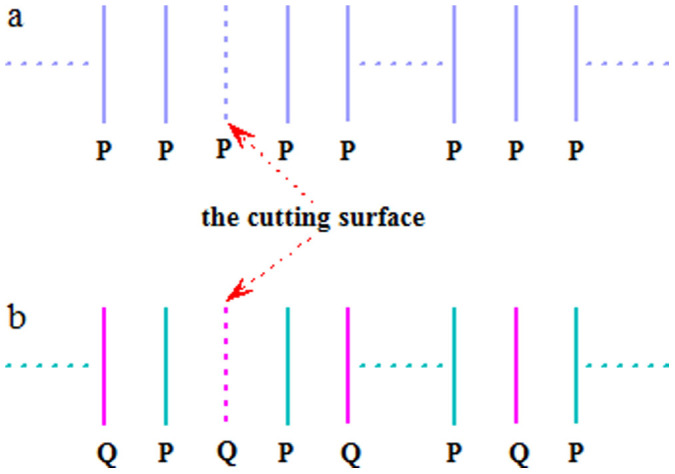
Two type structures for the crystals with the reflection symmetry. (a) Type I “…-*P*-*P*-*P*-*P*-…”. (b) Type II “…-*P*-*Q*-*P*-*Q*-…”.

The above conclusion also covers the case of 2D/1D crystals, in which the “surface” represents the atomic chain/point. In our following demonstration, the transfer matrix approach^30^,^31^ is applied. The crystals with the reflection symmetry are only one of two types: Type I: “…-*P-P-P*-*P-*…” in Fig. 1a and Type II: “…-*P-Q-P-Q-*…” in Fig. 1b where *P* and *Q* represent CPs. The same *P* (*Q*) represents exactly the same CP while *Q* ≠ *P* means that *P* and *Q* are different CPs. The bar “-” roughly describes the distance between the 1NN CPs. The same “-” means the same distance. Since Type II can be transformed into Type I by simple mathematical calculations, thus we at first concentrate our attention on the proof of Type I and then turn back to Type II.

Before presenting the proof of the above conclusion, we provide a definition of “surface state” at first. Surface states such that they propagate along the direction of the boundary surface and their amplitudes decay exponentially in distance normal to the boundary surface. In terms of the transfer matrix language, the surface state is defined by the following decay relation:
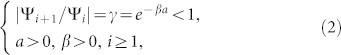
where *β* is a decay rate and *a* is the distance between the 1NN CPs. When γ = 1 (*β* *= * 0), it corresponds to the extended mode and must associate with the bulk state. Furthermore, since our model Hamiltonian is general, it also includes some particular case that can consist of two decoupled sub-lattices ***A*** and ***B***. Then Hamiltonian **H** can be decomposed into two decoupled parts: H = H*_A_* + H*_B_* Meanwhile we also assume that the sub-lattice ***B*** is not only decoupled from ***A*** H*_AB_ = * 0 but it also has no coupling among CPs. Then we have 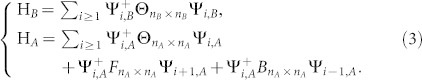
Thus 
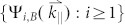
 are localized in the *i^th^* CP and have no propagation among CPs. Such states, even like 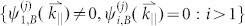, should be excluded from surface states. Here we focus on surface states such that their amplitudes decay exponentially in distance from the boundary plane.

### Proof for Type I

For the simplest case, each CP has only single electron mode that corresponds to one atomic orbit per unit cell. At the level of the 1NN hopping approximation, the study of surface states in this case is exactly the same as that of edge states in the semi-infinite 1D single orbit atomic chain. As is well known, no edge states exist in the semi-infinite 1D atomic chain for both Type I and II when the forward hopping constant equals to the backward one^31^. Thus, we will take into account the case that each *P* contains *n* (>1) electron modes. For a cut crystal “*P-P-P-P-…*” in Fig. 1a, it is not difficult to obtain following quantum dynamical equations (QDEs):
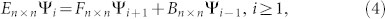
where *E_n ×n_* = diag{*E*_1_, *E*_2_,…, *E_n_*} and {*E_α_* = *E − ω_α_* : *α* = 1,2,…, *n*} *E* is the eigen energy of electron waves propagating in the crystal. {*ω_α_* : *α* = 1, 2,…, *n*} are energies of eigen-modes renormalized at each CP. The elements of *F_n ×n_, B_n ×n_* and {*ω_α_* : *α* = 1,2,…, *n*} are 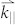 dependent. When the original infinite crystal has the reflection symmetry for each CP, we have *F_n ×n_, B_n ×n_*. Eq.(4) can be rewritten as
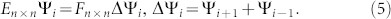
The matrices *F_n ×n_* and *E_n ×n_* are hermitian with the dimensionality *n*. Here we adopt the dimensional reduction method to reduce the dimensionality *n* in Eq.(5) to 1. We will prove that no surface waves accommodate in such a cut crystal for any energy *E*. Since we are not sure that *E_m ×m_* and F*_m ×m_* (*m < n*) are always hermitian in the dimensional reduction process, here what we assume is that *E_n ×n_* and *F_n ×n_* are arbitrary square matrices and are not limited to be hermitian from the beginning so that the following demonstration can be used repeatly in the dimensional reduction. But it is still applicable to the hermitian *F_n ×n_* and *E_n ×n_*.

### Proof for *n* > 2 in Type I

By means of the dimensional reduction method, we will reduce the dimensionality *n* in Eq.(5) into 1 or 2. Let us first consider an energy such that *E*: det(*E_n ×n_*) ≠ 0. Since det(*E_n ×n_*) ≠ 0, we can obtain from Eq.(5)
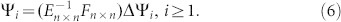
In the matrix theory, it is known that a square matrix 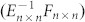 can be decomposed into a Jordan matrix via a similarity transformation 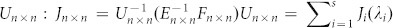 where *J_i_*(*λ_i_*) is the Jordan canonical block and its form is
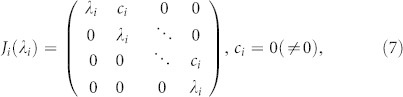
where the parameter *c_i_* = 0 or *c_i_* ≠ 0 depends on whether the matrix 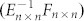 is a diagonalizable one or not and *λ_i_* is the eigenvalue of 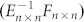. Now we have
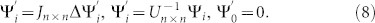
In terms of the property of the Jordan matrix *J_n ×n_* and from Eq.(8), we can reach immediately for 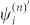

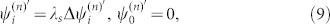
where *λ_s_* describes the effective coupling of the *n^th^* eigen mode between the 1NN CPs. Eq.(9) is exactly the same as the transfer matrix equation of 1D atomic chain with the single electron mode. It has been known that there are no edge states for any energy *E* no matter whether *λ_s_ = * 0 or *λ_s_* ≠ 0^31^. Thus we arrive at 
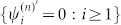
 for surface states. After back-substituting 
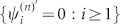
 into Eq.(8), we find that surface states are also impermissible for the (n − 1)^th^ mode, yielding 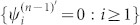 After step by step, we obtain 
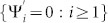
 for surface states that result in 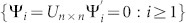 Hence no surface states are allowed for det(*E_n ×n_*) ≠ 0.

Next let us think over some energy such that *E*: det(*E_n ×n_*) = 0. Now we apply a Jordan transformation *V_n ×n_* to the matrix 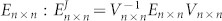 and have
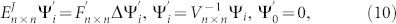
where 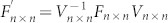. 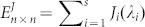 where we have arranged such that the sub-matrix *J*_1_ contains *λ*_1_ = 0. Without loss of generality, here we can assume the first block is a two-order Jordan sub-matrix at first. For other cases where the order of *J*_1_(*λ*_1_ = 0)is one or greater than two, we can do similar demonstrations as we do for a two-order Jordan block. The derivation can proceed by considering two scenarios:

1. Suppose 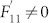. We can obtain from the first row of Eq.(10) 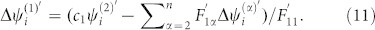
Substituting Eq. (11) into Eq. (10), we can arrive at 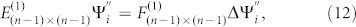
where 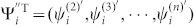 and 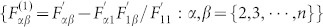. Thus, we have reduced the dimensionality *n* in Eq.(10) into *n−*1.
2. Next suppose 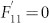. Now we focus on the first column matrix elements of the matrix 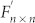. If they are all zero, the reduction of the dimensionality in Eq.(10) is already reached. Thus what we assume here is that there exists some *β* such that 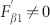 and *β ≠* 1 then we have 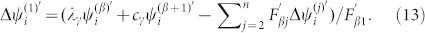 After substituting Eq.(13) into Eq.(10), we can get 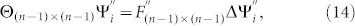
where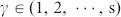 and 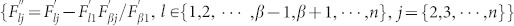. Elements in the matrix Θ_(*n*−1) × (*n*−1)_ are functions of energies {*E_α_* : α = 1,2,…, *n*} and hopping constants. 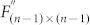 is a reduced effective hopping matrix and depends on the energy *E*. As a result, the dimensionality *n* in Eq.(10) has been reduced to *n*−1. If the determinant of 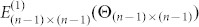 is nonzero, we can follow the similar steps from Eq.(6) to Eq.(9) to prove the absence of surface states. If the determinant of 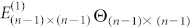 is zero, we will continue to reduce the dimensionality *n*−1 in Eq.(12) (Eq.(14)) to *n*−2 by means of the similar steps from Eq.(10) to Eq.(14). If necessary, we can do more reductions similar to above and eventually reduce the dimensionality in Eq.(10) to 1 or 2. Meanwhile, we can see that other modes 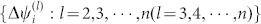 are either the linear combinations of 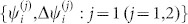 or can be decoupled as local modes when the dimensionality in Eq.(10) is reduced to 1(2). No surface states exist for the dimensionality 1 (as well known) when the forward hopping constant is equal to the backward one, neither for the dimensionality 2, as will be proved in the following.

### Proof for *n* = 2 in Type I

When *n* = 2, Eq.(5) is rewritten as
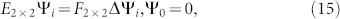
where 
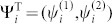
 and assuming *E*_2×2_ and *F*_2×2_ are general matrices in order to cover the previous case where the dimensionality in Eq.(10) is reduced to 2 when *n* > 2 and det(*E_n ×n_*) = 0. To ensure the validity of the proof for any energy *E* and any crystal structures, we must discuss all possible matrix structures of *E*_2 ×2_ and *F*_2 ×2._ At first, note that when det(*E*_2×2_) ≠ 0 or det(*F*_2 ×2_) ≠ 0 we obtain 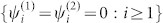 for surface states by the use of the similar steps from Eq.(6) to Eq.(9). Next, think over the special case where det(*E*_2 ×2_) = 0 and det(*F*_2 ×2_) = 0. We apply a Jordan similarity transformation *U*_2 ×2_ for *E*_2 ×2_ then Eq.(15) can be written as
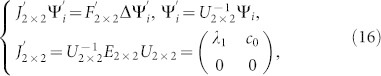
where 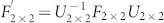 and {*λ*_1_, *c*_0_} can be zero or nonzero. We further examine the following three possible situations:

- *λ*_1_ = 0 and *c*_0_ = 0. We can apply the Jordan transformation again to 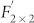 and since 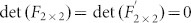, Eq.(16) becomes 
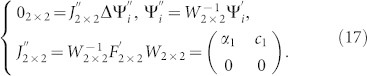
When *α*_1_ = 0 and *c*_1_ = 0, 
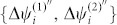
 fully decouple and become local modes within each CP. When *α*_1_ ≠ 0 and *c*_1_ = 0, 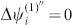 corresponds to an extended mode and 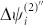 is decoupled as the local mode within each CP. When *α*_1_ = 0 and *c*_1_ ≠ 0, 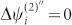 means the non-existence of surface states and 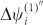 becomes the local modes without propagation among the CPs.
- *λ*_1_ = 0 and *c*_0_ ≠ 0. At first, we note that when 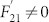 or 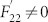, 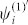 and 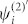 become local modes within each CP or are zero solutions for surface states. Next, consider the special case where 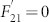 and 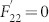, then we get from Eq.(16) 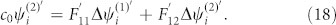
When 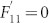, Eq.(18) turns into 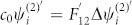 and we obtain 
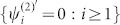
 for surface states. When 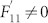, 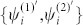 are coupled together. If there are surface states existing for 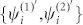, we can have 
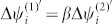
 where *β* is a non-zero constant. Then Eq. (18) becomes 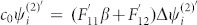 that results in 
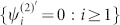
 for surface modes, leading to 
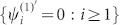
. Therefore, no surface states can exist in the cut crystal.
- *λ*_1_ ≠ 0 and *c*_0_ = 0. The proof is almost exactly similar to the case {*λ*_1_ = 0 and *c*_0_ ≠ 0 and we get the same conclusion. Up to now, the conclusion has been analytically proved for cut crystals with “*P-P-P-P-…*”.

### Proof for Type II

In Type II, the crystal has two different CPs: *P* and *Q*. We just discuss the *Q* cut crystal “*Q-P-Q-P-*…” in Fig. 1b since the discussion for the *P* cut crystal will be similar. Now the QDEs for the *Q* cut crystal are
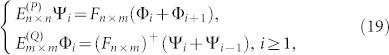
where {Φ*_i_* = 0*_m_*_×1_,Ψ*_i_* = 0_*n* ×1_ : *i* < 1} and the CP *P* has *n* modes and *Q* has *m* ones. *n* and *m* can be equal or unequal. 
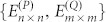
 are defined as 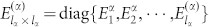, 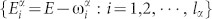 and *l_p(Q)_ =* *n*(*m*) when *α* = *P*(*Q*). After simple calculations, Eq.(19) can be written as
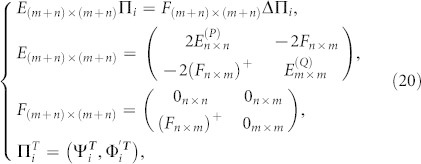
where 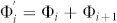 and ΔΠ*_i_* = Π*_i_*_+1_ + Π*_i_*_−1_. Now the *Q* cut crystal structure “*Q-P-Q-P-*…” in Type II is equivalent to “*P*′*-P*′*-P*′*-*…” in Type I with the dimensionality *m* + *n*. We can find 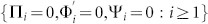 for surface waves. 
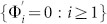
 yield Φ*_i_* + Φ*_i_*_+1_ = 0 : *i* ≥ 1 that further lead to {Φ*_i_* = 0 : *i* ≥ 1} for surface states. Hence the conclusion is also valid for Type II. So far, we have completed the proof of the conclusion for Type I and II. From the above demonstration, we clearly know that *F_n ×n_* = *B_n ×n_* is the key to the conclusion.

Our conclusion is only valid at the level of the hopping approximation between the 1NN CPs. In some real materials, although the longer range hoppings among the *n*NN (*n* > 1) CPs may be weak for some materials with low-index surfaces, they always exist. Meanwhile, the surface of real cut materials is imperfect and the surface relaxation is always unavoidable near the surface, even surface reconstruction. Thus here we study the effect of both factors on our conclusion.

### Effects of the longer range hopping and surface relaxation

At first, we focus on the effect of the longer range hoppings among the *n*NN (*n* > 1) CPs in the absence of surface relaxations. When they are much weaker than that between the 1NN CPs for the crystals with low-index surfaces, their effect can be estimated in terms of the perturbation theory. In the case, QDEs for a cut crystal with Type I can be written as
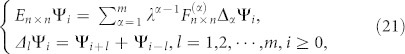
where {Ψ_i_ = 0 : *i* < 0} and *λ* characterizes the order of the small perturbation. The boundary surface is set at the zeroth layer. The terms 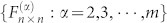 result from the longer range hopping among CPs. 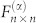 represents the *n* × *n* hopping matrix from one CP to its α*^th^* neighbor CP. According to the gradual development spirit of the perturbation theory, we can set
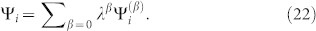
Inputting Eq.(22) into Eq.(21), we obtain
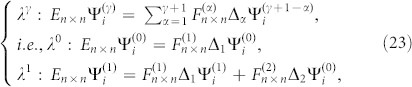
where {*β*, *γ = * 0, 1, 2 }. From Eq.(23), we can obtain 
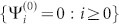
 for surface states from the above discussion. After back-substituting 
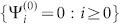
 into Eq.(23), we get 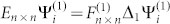. As demonstrated above, we can reach 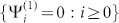. After step by step, we also can get 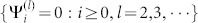 and further have {Ψ*_i_* = 0 : *i* ≥ 0} for surface states. Thus, surface states in crystals with the reflection symmetry are not allowable when they are much weaker than that between the 1NN CPs. When *λ* becomes large, the perturbation theory will be invalid and as a result surface states can emerge even if the crystal has the reflection symmetry (see the example of armchair edged graphene shown in the following section). In practice, the longer range hoppings within the CP may be large, but they are usually small among CPs for some crystals with low-index surfaces and can be regarded as a perturbation.

It is similar to consider small surface relaxations. Here we assume there are surface relaxations from 0^th^ to (*j* − 1)^th^ layer CP and energies of eigen modes renormalized at each CP are unchanged at the level of the hopping approximation between the 1NN CPs, then we obtain QDEs
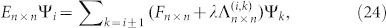
where 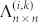 is from the small surface relaxation between the 1NN CPs and non-zero matrix when 0 ≤ *i* ≤ *j*. We input Eq.(22) into Eq.(24) and receive 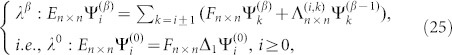
where {*β* = 0, 1, 2, … } and 
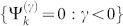
. As it has been done in the absence of surface relaxations, we can obtain 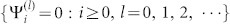 step by step. Thus, the small surface relaxation has no impact on our conclusion. So far, it is known that the weak longer range hopping and small surface relaxation cannot influence our conclusion, but they have the effect on the bulk states.

## Discussion

**Figure 2:**
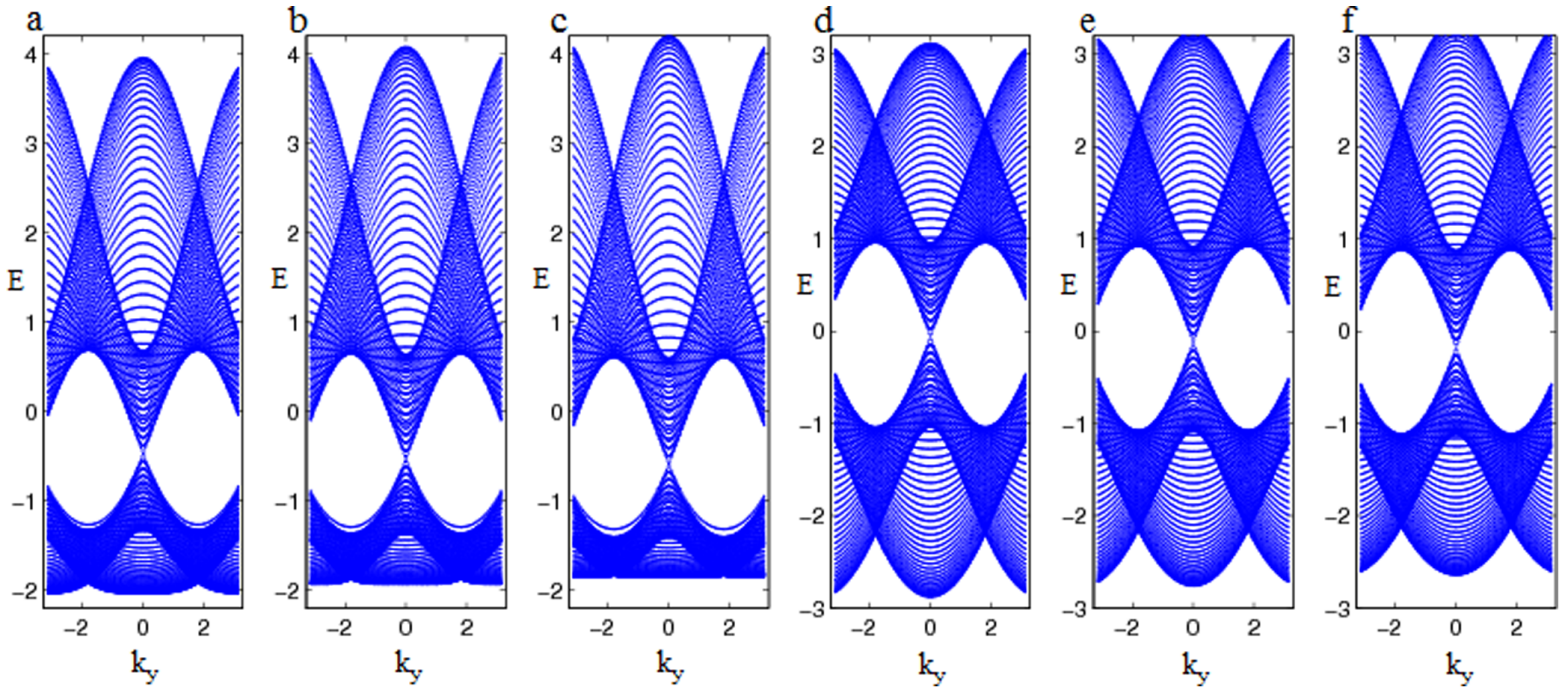
Energy spectrum of the armchair edged graphene with the 1NN hopping *t*_1_ and the different 2NN hopping *t*_2_. Note that surface states emerge in figures (a, b, c), while figures (d, e, f) show the absence of surface states. (a) *t*_2_ = 0.16*t*_1_. (b) *t*_2_ = 0.18*t*_1_. (c) *t*_2_ = 0.2*t*_1_. (d) *t*_2_ = 0.02*t*_1_. (e) *t*_2_ = 0.04*t*_1_. (f) *t*_2_ = 0.06*t*_1_.

**Figure 3:**
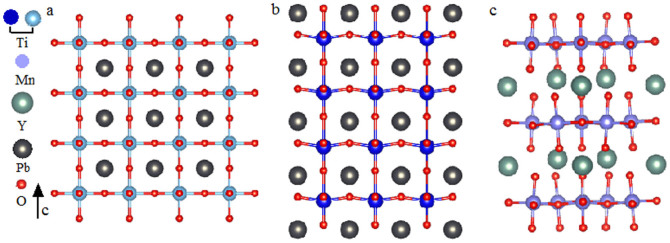
Crystal structures of ABO_3_ materials at the para-electric and ferroelectric phases. (a) c-cut PbTiO_3_ at the para-electric phase. (b) c-cut PbTiO_3_ at the ferroelectric phase. (c) c-cut YMnO_3_ at the ferroelectric phase.

In application of the conclusion, we can give some examples to support our demonstration and see its availability for qualitatively predicting the presence or absence of surface states in some materials. Firstly, we can easily check armchair edged graphene with the 1NN hopping *t*_1_ only has no edge states since it has “*P-P-P-P*-…” type structure, consistent with the previous theoretical analysis^32^. We also study the effect of the 2NN hopping *t*_2_ on surface states in terms of the exact diagonalization method where *t*_2_ is taken in the region 0.02*t*_1_ ≤ *t*_2_ ≤ 0.2*t*_1_^33^,^34^ and find that surface states can appear for the ratio 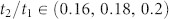 in Fig. 2a–c, but not for 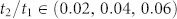 in Fig. 2d–f. Thus the longer range hopping among CPs is able to induce surface states if it is strong enough and not regarded as a perturbation, compatible with our demonstration. Furthermore, the type structure of zigzag edged graphene is “*P = P-P = P-*…” where the *F-B* symmetry is broken, thus it is in favor of the existence of edge states, shown in the zero mode result^31^. Secondly, let us further address ABO_3_ Perovskites since they are very much important in device applications. Ab initio calculations of ABO_3_ Perovskite (001) surfaces have shown no surface states^24^,^25^. The SOC and/or correlation effect in these materials do not play an important role and as a result they are suitable for our model. From the structure symmetry analysis of ABO_3_ Perovskites such as PbTiO_3_, we predict that the semi-infinite c-cut ABO_3_ in the para-electric phase have no surface states at the level of the 1NN hopping approximation since its type structure is “*P-Q-P-Q-*…” in Fig. 3a, consistent with ab initio calculations^24^,^25^. But we can find that surface states may appear in the above c-cut ABO_3_ with the polarization along the c-axis since it breaks the *F-B* symmetry in Fig. 3b. Another example is the c-cut ferroelectric YMnO_3_ in Fig. 3c which has the hexagonal structure. It has surface states due to the *F-B* asymmetry, compatible with the previous study^21^.

In conclusion, based on lattice model Hamiltonian with the hopping between the 1NN CPs and all possible neighbor hoppings within each CP, we have proved that there will not be surface states in a (hkl) cut crystal if the original infinite crystal has the reflection symmetry about each (hkl) CP. Meanwhile we also consider the effect of the longer range hoppings among the *n*NN (*n* > 1) CPs and surface relaxations on our conclusion. For some types of crystals (not like ionic crystals) with low-index surfaces, the longer range hoppings are weak enough and can be regarded as a perturbation, then they have no effect on our conclusion. It is also shown that small surface relaxations have no impact on our conclusion. In fact, the *F-B* dynamical symmetry (F_*n ×n*_ = B_*n ×n*_) is the key in our demonstration. For the crystals without SOC and/or strong correlation effect, we find that different cutting surfaces of the same crystals may have the different behavior for the existence of surface states. Moreover, our proof can be extended to *F_n ×n_* = *e^iδ^B_n ×n_* where *δ* is a *k*-dependent or zero. Our model in the above demonstration, in some sense, is much closer to real materials than previous simple ones. Thus our conclusion may be helpful to visually predict which cutting direction of the crystals is unfavorable for generating surface states in future research. Finally, we would also mention that our model is limited. In practice, surface states can exist on low index metal surfaces of FCC and BCC elemental metals such as Ag, Nb and Fe where SOC and/or strong correlation effect are/is dominant^35^,^36^,^37^. Thus our model is invalid for the crystal with strong SOC or/and strong correlation effect, such as TI and TSC. Although our conclusion may have the potential for predicting the absence of surface states in some cut crystals, a criterion for the presence of surface states still remains to be investigated.

## Methods

We started from a lattice model Hamiltonian at the level of effective single-particle approximation and analytically proved that there should not be surface states in a cut crystal with *F-B* symmetry. The Jordan matrix property and transfer matrix approach were also used to reduce the dimensionality of QDEs. Furthermore, we also extend our method to present the more general argument for the effect of the coexistence of the weak longer range hopping and small surface relaxation. What we assume is that there exist small relaxations from 0^th^ to (j − 1)^th^ layer CP and the weak longer range hoppings among the *n*NN CPs (2 ≤ n ≤ m) and energies of eigen modes renormalized at each CP are slightly changed due to atomic relative movement at each CP, then we can obtain QDEs
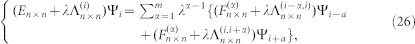
where {Ψ*_i_* = 0 : *i* < 0} and 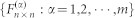 come from the coupling between the *αNN* CPs. 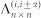 is from the small surface relaxation between the *αNN* CPs and 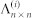
results from small relaxations within each CP. Substituting Eq.(22) into Eq.(26), we obtain
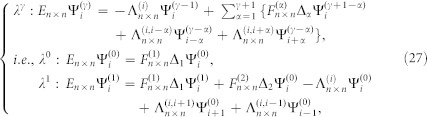
where 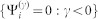. As demonstrated in the absence of both factors, we can arrive at 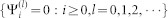 step by step and find that the small perturbation from both factors has no impact on our conclusion.

## Author Contributions

T.G. did the demonstration for the system with two electron modes only. H.W. and R.T. extended the model to the general case and developed dimensional reduction method to finish the general demonstration including the effect of the weak longer range hoppings and/or small surface relaxations. H.W. and R.T. wrote the manuscript. All authors discussed the results and commented on the manuscript.
